# Fibrin clot structure remains unaffected in young, healthy individuals after transient exposure to diesel exhaust

**DOI:** 10.1186/1743-8977-7-17

**Published:** 2010-06-16

**Authors:** Sofian Metassan, Michael N Routledge, Andrew J Lucking, Shirley Uitte de Willige, Helen Philippou, Nicholas L Mills, David E Newby, Robert AS Ariëns

**Affiliations:** 1 Division of Cardiovascular and Diabetes Research, Section on Mechanisms of Thrombosis, Leeds Institute for Genetics Health and Therapeutics, Faculty of Medicine and Health, University of Leeds, Leeds, UK; 2 Molecular Epidemiology Unit, Leeds Institute for Genetics Health and Therapeutics, Faculty of Medicine and Health, University of Leeds, Leeds, UK; 3 Centre for Cardiovascular Science, University of Edinburgh, Edinburgh, UK

## Abstract

Exposure to urban particulate matter has been associated with an increased risk of cardiovascular disease and thrombosis. We studied the effects of transient exposure to diesel particles on fibrin clot structure of 16 healthy individuals (age 21- 44). The subjects were randomly exposed to diesel exhaust and filtered air on two separate occasions. Blood samples were collected before exposure, and 2 and 6 hours after exposure. There were no significant changes on clot permeability, maximum turbidity, lag time, fibre diameter, fibre density and fibrinogen level between samples taken after diesel exhaust exposure and samples taken after filtered air exposure. These data show that there are no prothrombotic changes in fibrin clot structure in young, healthy individuals exposed to diesel exhaust.

## Findings

Exposure to urban particulate matter (PM) air pollution has been associated with increased risk of cardiovascular disease and mortality [1]. Peters *et al*. showed that exposure to PM has an immediate effect on acute cardiovascular events [2], with a peak in admission to hospital within as little as 2 hours after exposure. Hamster models showed increased arterial and venous thrombosis less than 1 hour after exposure to diesel exhaust [3]. In agreement with this, human subjects who were transiently exposed to dilute diesel exhaust had increased *ex vivo *thrombus formation [4]. A study by Baccarelli *et al*. showed hypercoagulability after transient PM exposure [5]. These studies suggest prothrombotic effects due to short term exposure to PM.

*In vitro *and *in vivo *data suggest that the biological effects of PM are mostly attributable to ultrafine particulate matter (UFPM) with an aerodynamic diameter of less than 100 nm [6-8]. These particles have a large surface area per unit mass and are therefore capable of carrying larger amounts of harmful compounds on their surface [9]. Furthermore, some reports suggested that UFPM may be translocated into the circulation either passively or by active transport mechanisms [10,11]. Detectable levels of UFPM have been found in the liver, kidney, spleen and heart of exposed animals [6,12,13]. Exposure to PM, and UFPM in particular, has been associated with systemic inflammation and increased blood coagulability [5,14-16].

Coagulation produces a fibrin clot which has three-dimensional network characteristics. The structure of the fibrin network is an important determinant of clot rigidity, susceptibility to fibrinolysis and cell interactions. We recently found that *in vitro *addition of PM to fibrinogen changes clot structure producing a heterogeneous network with densely knit fibrin interspersed with looser areas [17]. We therefore tested the hypothesis that transient exposure to diesel exhaust may lead to changes to fibrin structure and function *in vivo*.

Sixteen healthy subjects aged 26 ± 5 (mean ± SD, range 21- 44) were randomly exposed to filtered air or dilute diesel engine exhaust (PM: 350 μg/m^3^) for 2 hours performing moderate exercise on a bicycle for 15 min alternated with 15 min rest periods. This exposure level is regularly reached during rush-hour traffic in large cities [18]. The study was approved by the local research Ethics Committee (Edinburgh), in accordance with the Declaration of Helsinki. Each subject agreed to participation in the study by written informed consent [4]. The study had a double-blind cross-over design, hence the same subjects, unaware of the exposure type, were exposed to air on one occasion and to diesel on another (minimum time between the exposures was 2 weeks). Blood was collected on citrate before and 2 and 6 hours after exposure. Platelet poor plasma was prepared by centrifugation and samples were stored below -40°C.

Fibrin clot permeation analysis was performed on clots made from plasma by the addition of 1 U/ml thrombin and 10 mM CaCl_2_. Permeation was performed and the permeation constant Ks, which describes average pore-size in cm^2^, was calculated as described [17,19]. Fibrin polymerisation was investigated by optical density (OD) measurements at a wavelength of 340 nm every 12 seconds in a 96 well microtiterplate using an ELx808 BioTek microplate reader (Winooski, VT, USA). Plasma was diluted 6-fold and clot formation was initiated by addition of 0.03 U/ml thrombin and 10 mM CaCl_2_. Lag time (time to increase in OD of > 0.01) and maximum OD (ODmax) were measured.

Fibrin clots for laser scanning confocal microscopy were made in a μ-slide VI (Ibidi, Germany) by the addition of 0.5 U/ml thrombin and 10 mM CaCl_2 _to plasma (diluted 2-fold in 0.05 M Tris-HCl, 0.1 M NaCl, pH 7.4). The slide was kept in a moist chamber and confocal microscopy was performed on a Leica TSC-SP2 as described [17,20]. Fibre diameters were analysed using the Leica software. Fibre density was calculated as the number of fibres crossing a straight line of fixed length across the scanfield.

Fibrinogen levels were measured in plasma using ELISA. Rabbit anti-human fibrinogen IgG (DAKO A0080), diluted 1/8000, was used as capture antibody. Bovine serum albumin (1% [w/v]) was used to block non-specific sites. Plasma (diluted 1/128,000 and 1/256,000) was subsequently incubated for 1 hour. Goat polyclonal anti-fibrinogen HRP-conjugated IgG (Abcam ab7539), diluted 1/16,000 was used for detection. The assay was developed using OPD tablets (DAKO). All incubations were carried out at room temperature. Fibrinogen levels were calibrated against a standard curve (1.56 ng/ml to 25 ng/ml) of purified human fibrinogen (Calbiochem, UK).

For each subject, we calculated the change in fibrin parameters from baseline to 2 hours after exposure and from baseline to 6 hours after exposure. Paired t-test was used to test whether changes in fibrin parameters were different between exposure to air and exposure to diesel. P-values less than 0.05 were taken as statistically significant.

We analysed a total of 93 samples. For one subject, samples were unavailable after diesel exhaust exposure. Table 1 summarises the data obtained. Compared to before exposure, maximum turbidity (ODmax, indicative of average fibre mass-length ratio) was significantly increased 2 hours after exposure to filtered air (p = 0.032). However, this effect was not seen 2 hours after exposure to diesel exhaust. There was a decrease in lag time 6 hours after diesel exhaust exposure, but this value did not differ significantly from either the pre-exposed value (p = 0.082) or from the lag time 6 hours after filtered air exposure (p = 0.56). There were no significant changes in Ks (analysis of fibrin pore-structure by permeability), or in fibre diameter and fibre density as analysed by confocal microscopy after exposure to both diesel exhaust and filtered air. Fibre density is inversely proportional to Ks and its lack of changes after diesel exhaust is in agreement with the permeability measurement. Fibrinogen level was significantly decreased 6 hours after exposure to filtered air (p = 0.025). However, this fall in fibrinogen level was not significantly different compared to fibrinogen levels 6 hours after diesel exhaust exposure (p = 0.26).

**Table 1:** Fibrin clot structure in young, healthy volunteers exposed to filtered air and diesel exhaust

						P values for comparison of changes between filtered air and diesel exhaust	
	**Pre-exposed**	**2 hr**	**6 hr**	**Δ 2 hr**	**Δ 6 hr**	**Δ 2 hr**	**Δ 6 hr**

**Filtered air (n = 16)**							
**Ks (×10**^**-9 **^**cm**^**2**^**)**	5.17 ± 1.48	5.27 ± 2.64	5.13 ± 1.22	0.10 ± 2.01	-0.04 ± 0.92	-	-
**ODmax**	0.230 ± 0.051	0.255 ± 0.078	0.233 ± 0.059	**0.025 ± 0.042**	0.003 ± 0.021	-	-
**Lag time (seconds)**	917 ± 321	906 ± 352	922 ± 482	-11 ± 302	5 ± 385	-	-
**Fibre Diameter (nm)**	458 ± 18	457 ± 18	470 ± 25	-1 ± 18	12 ± 24	-	-
**Fibre Density (fibres/120 μm)**	7.3 ± 1.6	7.5 ± 1.6	7.2 ± 1.8	0.2 ± 1.1	-0.1 ± 2.1	-	-
**Fibrinogen level (mg/ml)**	2.05 ± 0.43	2.12 ± 0.43	1.81 ± 0.31	0.07 ± 0.48	**-0.24 ± 0.36**	-	-

**Diesel Exhaust (n = 15)**							
**Ks (× 10**^**-9**^**cm**^**2**^**)**	4.94 ± 1.89	5.44 ± 1.46	4.85 ± 1.26	0.51 ± 1.95	-0.09 ± 1.32	0.55	0.90
**ODmax**	0.246 ± 0.044	0.244 ± 0.039	0.238 ± 0.039	-0.002 ± 0.024	-0.008 ± 0.036	0.03**§**	0.17
**Lag time (seconds)**	846 ± 223	788 ± 226	737 ± 201	-58 ± 270	-109 ± 226	0.64	0.56
**Fibre Diameter (nm)**	455 ± 21	459 ± 16	465 ± 21	4 ± 21.7	10 ± 24.4	0.54	0.91
**Fibre Density (fibres/120 μm)**	7.8 ± 1.4	8.1 ± 1.7	8.1 ± 1.7	0.3 ± 1.2	0.3 ± 1.2	0.97	0.59
**Fibrinogen level (mg/ml)**	2.10 ± 0.45	2.08 ± 0.41	1.96 ± 0.22	-0.02 ± 0.33	-0.14 ± 0.32	0.76	0.26

Data represents before exposure (pre-exposed), 2 hours after exposure (2 hr), 6 hours after exposure (6 hr), the difference between 2 hours and pre-exposed (Δ 2 hr) and the difference between 6 hours and pre-exposed (Δ 6 hr). The values are mean ± standard deviation. P values were calculated by paired t-test.

Bold typeset indicates values that were significantly different from baseline.

**§ **indicates the changes after diesel exhaust exposure were significantly different from those after filtered air exposure.

Fibrin is formed at the site of damaged blood vessels and its structure is an essential determinant of the elastic properties of the clot and susceptibility to fibrinolysis. Fibrin clots with decreased Ks (as a result of tighter fibrin networks), increased fibre density and thinner fibres are commonly found in patients with acute coronary syndrome [21,22]. Since exposure to UFPM is associated with cardiovascular disease, we hypothesised that exposure to diesel exhaust leads to altered fibrin clot structure. In addition, we recently observed changes in fibrin clot structure upon exposure to PM *in vitro*. However, here we find that fibrin properties in plasma from volunteers after transient diesel exhaust exposure remained remarkably similar to those in the samples of the same subjects after exposure to filtered air, showing that at least in healthy, young individuals fibrin structure is not affected by PM exposure at typical environmental exposure levels.

Due to the transient nature of the diesel exposure in this study, we cannot predict the effects of more chronic exposure to pollution on fibrin clot structure. Chronic exposure to PM likely precipitates and aggravates pulmonary disease and existing cardiovascular disease. People chronically exposed to high PM concentration have been shown to have a greater risk of pulmonary and cardiovascular diseases compared to those living in less polluted areas [23]. As our study population involved young, healthy subjects, we cannot predict the effect of exposure to PM on fibrin structure in susceptible individuals such as the elderly, diabetic patients and patients with chronic obstructive pulmonary disease [24]. It would also be of interest to examine the effect of higher occupational exposures (e.g. bus garage workers) on fibrin clot structure. Based on our data we conclude that transient exposure to diesel in young, apparently healthy individuals is not associated with changes in fibrin clot structure.

## List of abbreviations used

ELISA: enzyme-linked immunosorbent assay; HRP: Horseradish peroxidise; IgG: immunoglobulin; Ks: Darcy or permeation constant, measure of the pore-size in fibrin clots; ODmax: maximum optical density reached in turbidity assays, indicative of average fibre mass-length ratio; OPD: o-phenylenediamine dihydrochloride; PM: particulate matter; UFPM: ultrafine particulate matter

## Competing interests

The authors declare that they have no competing interests.

## Authors' contributions

SM performed the fibrin structure analysis and fibrinogen measurements and wrote the manuscript. MNR designed the study and provided critical comments. AJL, NLM and DEN designed and performed the transient diesel exposure study and provided critical comments. SUdW helped with the fibrinogen measurements and statistical analysis and provided critical comments. HP helped with experimental design and provided critical comments. RASA designed the study and wrote the manuscript. All authors read and approved the final manuscript.
